# Microbial composition of tumorous and adjacent gastric tissue is associated with prognosis of gastric cancer

**DOI:** 10.1038/s41598-023-31740-3

**Published:** 2023-03-21

**Authors:** Konrad Lehr, Darja Nikitina, Ramiro Vilchez-Vargas, Ruta Steponaitiene, Cosima Thon, Jurgita Skieceviciene, Denny Schanze, Martin Zenker, Peter Malfertheiner, Juozas Kupcinskas, Alexander Link

**Affiliations:** 1 Department of Gastroenterology, Hepatology and Infectious Diseases, Section of Molecular Gastroenterology and Microbiota-associated Diseases, Otto-von-Guericke University Magdeburg, Magdeburg, Germany; 2 Institute for Digestive Research, Lithuanian University of Health Sciences Kaunas, Kaunas, Lithuania; 3 Institute of Human Genetics, Otto-Von-Guericke University, Magdeburg, Germany; 4 Department of Internal Medicine 2, University Hospital, LMU Munich, Munich, Germany; 5 Department of Gastroenterology, Lithuanian University of Health Sciences Kaunas, Kaunas, Lithuania

**Keywords:** Gastroenterology, Cancer microenvironment, Gastrointestinal cancer, Translational research, Microbiome

## Abstract

*Helicobacter pylori* (*H. pylori*) infection has been considered as the main causal factor in gastric carcinogenesis, but other bacterial species may also play an important role in pathophysiology of gastric cancer. The aim of the study was to explore the link between gastric cancer prognosis and the mucosal microbial community in tumorous and adjacent gastric tissue. The bacterial profile was analysed using 16S sequencing (V1–V2 region). Microbial differences were mostly characterized by lower relative abundances of *H. pylori* in tumorous gastric tissues. Bacterial community and outcome data analysis revealed the genus *Fusobacterium* and *Prevotella* significantly associated with worse overall survival in gastric cancer patients. In particular, *Fusobacterium* was associated with significant increase in hazard ratio in both univariable and multivariable analysis and independently validated using TCMA data. Phylogenetic biodiversity of *Fusobacterium* species in the stomach revealed *F. periodonticum* as the most prevalent in healthy subjects, while *F. nucleatum* was most abundant in patients with gastric cancer. Bacterial community network analysis in gastric cancer suggests substantial complexity and a strong interplay between *F. nucleatum* and *Prevotella.* In summary, mucosal microbial community in the stomach was associated with worse overall survival in gastric cancer patients. Strongest negative impact on prognosis was linked to the abundance of *F. nucleatum* in tumorous specimens, suggesting its translational relevance in management of gastric cancer patients.

## Introduction

Identification of *Helicobacter pylori* (*H. pylori*) in the stomach has been the initial step in stomach microbiome research since it supplanted the initial hypothesis of the stomach as a sterile niche due to its low pH^1^. It then took only a few years to demonstrate the causal link of *H. pylori* with gastric cancer (GC)^2^. Gastric mucosa is strongly assessable for the microbial long-term impact as *H. pylori* promotes gastric carcinogenesis through the cascade of molecular events along with multistep histopathological events such as chronic gastritis which may progress to atrophic gastritis, intestinal metaplasia, and finally to dysplasia and GC^3,4^.

Over the last years, various bacteria belonging to different phyla, such as Proteobacteria, Firmicutes, Actinobacteria, Bacteroidetes, and Fusobacteria, were discovered in the stomach mucosa^5^. Detailed analysis of the gastric microbiome revealed a high microbial diversity with at least 95 different genera present in the stomach habitat. Each individual harbours a unique composition of bacteria. Despite this high diversity, there are dominant bacterial genera in a healthy stomach like *Gemella*, *Veillonella*, *Neisseria*, *Fusobacterium*, *Streptococcus*, *Prevotella*, *Pseudomonas*, and *Actinomyces*^6–9^. Changes in the microbial community of the stomach are strongly driven by *H. pylori* positivity and less dependent on genetic factors^6^.

Overall, studies suggest that increasing amount of stomach bacteria may colonize the stomach mucosa and be associated with GC. Higher abundance of *Fusobacterium nucleatum*, *Clostridium colicanis*, and *Lactobacillus gasseri* or *Lactobacillus reuteri* have been linked to GC^10–12^. Preliminary studies have revealed that gastric microbiota in tumour tissue is characterized by reduced diversity, reduced *H. pylori* abundance and enrichment for intestinal commensals^13^. Microbial composition is nevertheless *H. pylori* dependent, and in subjects without *H. pylori* infection associated with enrichment of *Peptostreptococcus stomatis, Streptococcus anginosus, Parvimonas micra, Slackia exigua*, and *Dialister pneumosintes* along the GC progression^14^.

Some studies analysed the bacterial communities in faecal samples of patients with GC^15,16^. Faecal *Streptococcus* alterations have been linked to GC and liver metastases and have therefore been suggested as a potential diagnostic biomarker^17^. However, faecal samples, although easily accessible, offer only limited insight on the pathophysiological interaction between the gastric mucosa and microbiome^8,18,19^. Microbial GI tract mapping has recently revealed a distinct microbial community in the stomach in comparison to intestine and faeces^6,8^.

Mounting evidence has been gathered on *Fusobacterium nucleatum (F. nucleatum)*, which is among the most prevalent bacteria in CRC^20,21^. *F. nucleatum* is enriched in CRC primary tumour tissues and is associated with CRC progression, recurrence, and with worse prognosis in CRC^21,22^. Recently, using a targeted approach, we identified *F. nucleatum* positivity associated with worse overall survival (OS) in GC patients^23^. Despite this rapidly increasing knowledge linking gastric microbiota with gastric carcinogenesis, studies that use microbiome sequencing to evaluate the impact of microbial species on prognosis of GC patients are lacking.

**Figure 1:**
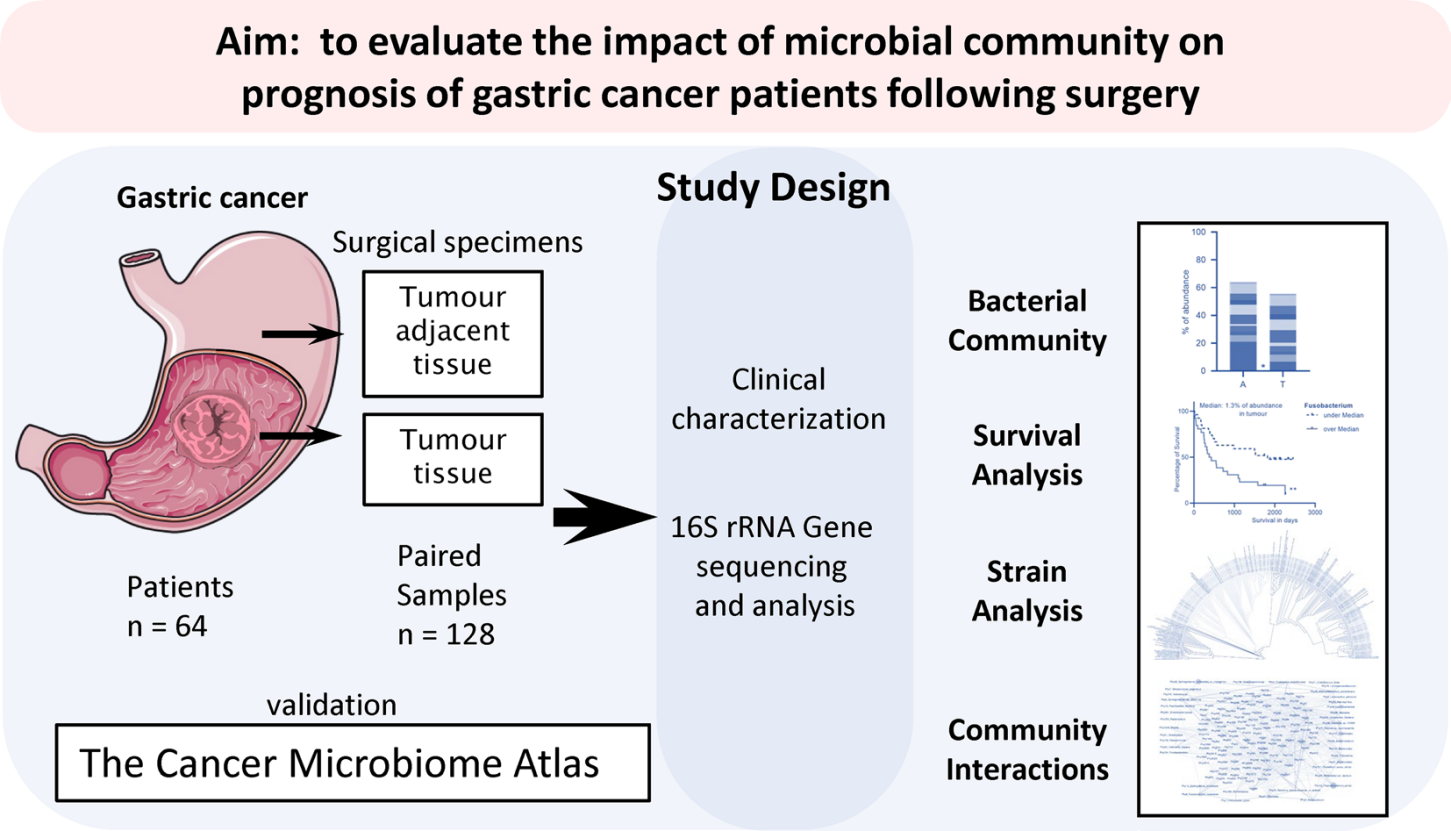
Graphical abstract of the study design.

The aim of our study was to unravel the potential association between the whole microbiome alterations from tumorous and adjacent tissues and the prognosis of GC patients. Furthermore, we performed in-depth analysis on phylogenetic biodiversity of identified species linked to prognosis of GC patients in comparison to non-GC populations (Fig. 1).

## Results

After sequencing, filtering, denoising, merging, chimeras removal, and normalizing to the minimum sequencing depth of 4153 sequences, the cohort was formed by 128 samples from 64 patients (each n = 64 tumorous GC tissue (T-GC) and non-tumorous GC (NT-GC)), from which roughly half a million reads were retrieved and merged into 5450 different phylotypes belonging to 19 phyla and 296 genera.

### Variation of the bacterial communities in the stomach of GC patients

**Figure 2:**
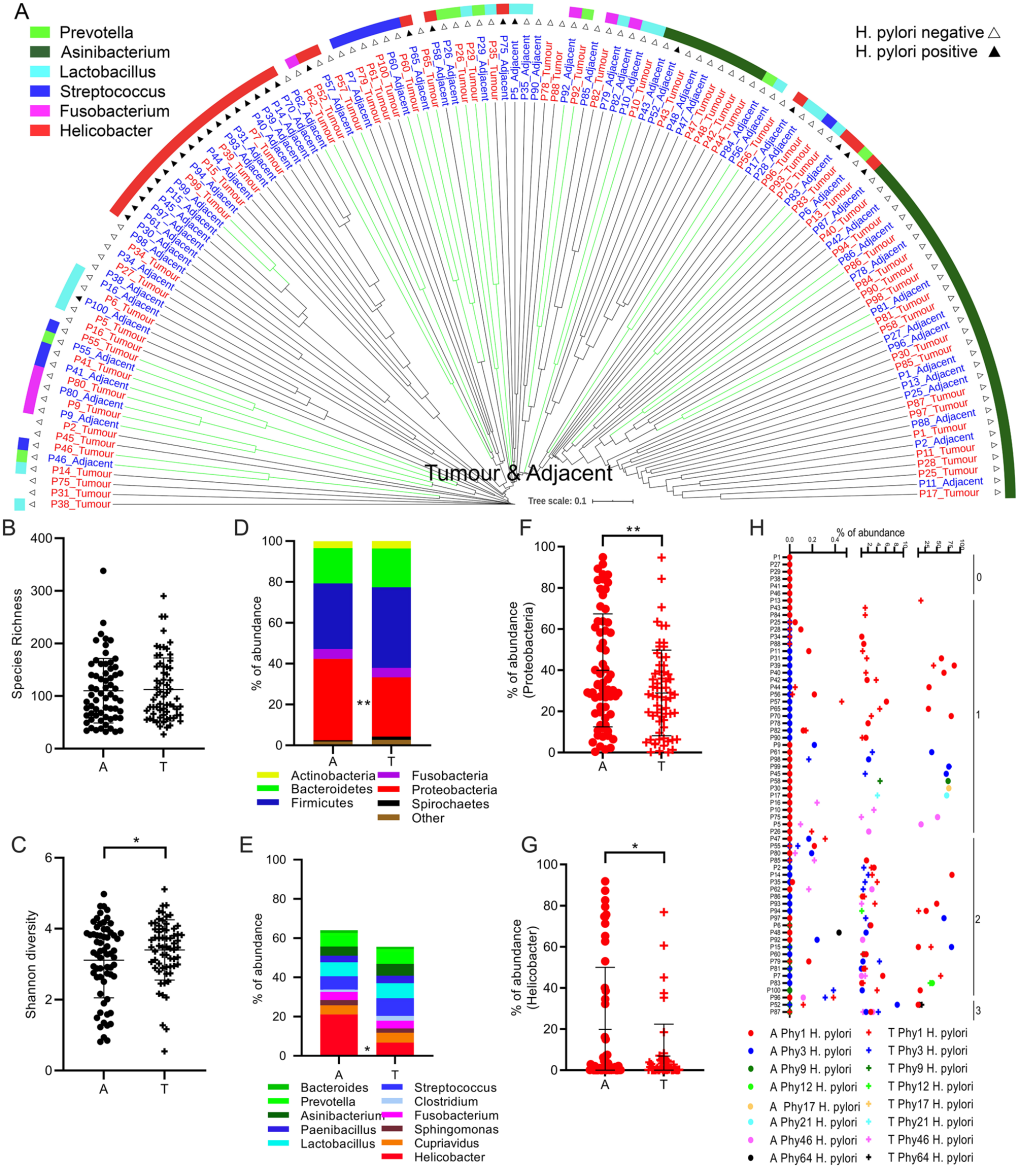
Overall bacterial communities based on the Bray–Curtis similarities between the samples of the complete cohort (**A**). Only the samples of the same patient that clustered together are shown in green lines. If the abundance of *H. pylori* was > 15% this is denoted with black filled triangle and if < 15% with black triangle. The most abundant genus found in the sample is displayed with different colours. Species richness (**B**) and Shannon diversity (**C**) of every adjacent and tumour tissue sample. Global bacterial community in adjacent tissue and in tumour tissue at phylum (**D**) and genus (**E**) level. Relative abundances of the phylum Proteobacteria (**F**) and the genus *Helicobacter* (**G**) in adjacent and tumour tissue. Significantly different taxa are indicated by * if q < 0.05 and ** if q < 0.01. Percentage of abundance of the phylotypes belonging to *H. pylori in* each patient (**H**). The patients are sorted as a function of the total number of different phylotypes detected (right).

First, we evaluated the differences in microbial composition of mucosa from NT-GC and T-GC specimens from GC patients. As shown in the Fig. 2A, we observed comparable bacterial community in 28% (18 out of 64) of paired T-GC and NT-GC specimens, while 72% differed between T-GC and NT-GC tissues. The Bray–Curtis similarity for the samples clustering together was 56 ± 14%, while for the remaining samples it was 22 ± 14%, suggesting a very high intra-individual variation of the bacterial communities within the stomach between NT-GC and T-GC. Due to this intra-individual variation, both PERMANOVA and ANOSIM analyses showed no statistical differences between the bacterial communities in T-GC and NT-GC at all taxonomic ranks (at phylotype level: *p* = 0.7, F = 0.9 and *p* = 0.5, R = 0, respectively). The data on phylotype variation and abundance are shown in Supplementary Fig. S1. Intra-individual variation was found in 46 patients, where the bacterial community from NT-GC and T-GC of the same patient differed considerably. Despite this variability, a dominant phylotype with at least 10% of abundance was detectable in all samples. It is worth mentioning that phylotypes which are typically found only in the lower GI^8,9^ were detected in the stomach tissues to a greater or lesser extent, such as *Bacteroides*, *Clostridium *sensu stricto, or *Escherichia*/*Shigella*, suggesting a possible shift in the colonization of microorganisms from the lower to the upper GI in patients with GC (Supplementary Tables S5 and S6).

### Characterization of bacterial community between tumorous and non-tumorous specimens

The species richness was overall comparable between NT-GC and T-GC samples (Fig. 2B). Analysis of the taxa distribution between the groups showed statistical difference only in the abundance of Proteobacteria (specifically *Helicobacter pylori)* between both tissues (Fig. 2D,E), with a greater abundance in NT-GC in comparison to T-GC (Proteobacteria q = 0.009 and *Helicobacter* q = 0.01). In correlation, the Shannon diversity was higher in T-GC than in NT-GC specimens (Fig. 2C), which is likely to reflect the *H. pylori*-linked changes. As expected, the variation of *H. pylori* abundance in both NT-GC and T-GC ranged from undetectable to highly abundant levels (Fig. 2F,G). In total, 8 different *H. pylori* phylotypes were recognized in *H. pylori* positive samples, while no phylotype was observed in 6 patients. Among subjects harbouring *H. pylori* sequences, 32 patients were infected with only one phylotype, 23 patients carried two phylotypes, and 3 patients were infected by three different phylotypes; however, more detailed characterization was not possible (Fig. 2H).

### Mucosal microbial composition and OS

The survival data of 53 GC patients (83% of the cohort) were available for up to seven years (Table 1). To evaluate the effect of various factors, including age, gender, tumour region, and tumour staging (UICC and Lauren’s classification), we first performed OS analysis regarding those factors. The OS analysis revealed no significant difference related to age, gender, and tumour localization (Supplementary Fig. S2A–C). In comparison, patients with advanced tumour stages (III–IV) and Lauren´s diffuse tumour type showed worse OS compared to those with earlier tumour stages (I–II) and intestinal type (*p* = 0.03 and *p* = 0.049), respectively (Supplementary Fig. S2D,E). No difference was observed in frequently discovered genera between major histological tumour types based on Lauren´s or UICC classification (Supplementary Fig. S3A,B). Lower values were observed for the Shannon index and for species richness in diffuse tumours compared to intestinal type GC tumours (q = 0.029 and q = 0.036, respectively) (Supplementary Fig. S3C–F).

**Table 1:** Clinicopathological characterization and univariable and multivariable analysis.

Varialbe	Characterization of the cohort			Univariable analysis			Multivariable analysis		
				HR	(95% CI)	*p* value	HR	(95% CI)	*p* value
Age	66 ± 12		Continuous	1.02	0.993 to 1.050	0.1559	1.007	0.964 to 1.053	0.7699
Gender	Female	28 (46%)	Male vs female	1.407	0.717 to 2.752	0.3151	1.059	0.447 to 2.411	0.8927
	Male	33 (54%)							
Localisation	Cardia	8 (12%)	Corpus vs Antrum	0.875	0.418 to 1.917	0.7277	0.512	0.164 to 1.520	0.2355
	Corpus	28 (44%)	Cardia vs Antrum	2.154	0.782 to 5.565	0.1186	0.741	0.108 to 3.914	0.7394
	Antrum	17 (27%)							
	Unknown	11 (17%)							
UICC	I–II	25 (39%)	I–II vs III–IV	**2.115**	1.068 to 4.414	**0.0366**	2.467	1.010 to 6.418	0.0531
classification	III–IV	31 (49%)							
	Unknown	8 (12%)							
Lauren's	Diffuse	32 (50%)	Intestinal vs diffuse	0.481	0.218 to 0.988	0.0548	**0.233**	0.089 to 0.555	**0.0017**
classification	Intestinal	16 (25%)							
	Mixed	5 (8%)							
	Unknown	11 (17%)							
*Prevotella*			Median	**1.96**	1.008 to 3.907	**0.0494**	1.42	0.561 to 3.751	0.4647
Asinibacterium			Median	1.447	0.748 to 2.845	0.274	1.11	0.36 to 3.772	0.8599
Lactobacillus			Median	1.59	0.818 to 3.166	0.1755	1.566	0.714 to 3.565	0.27
Streptococcus			Median	1.204	0.624 to 2.359	0.581	1.095	0.445 to 2.702	0.8423
*Fusobacterium*			Median	**2.426**	1.242 to 4.902	**0.0107**	**3.93**	1.454 to 11.43	**0.0087**
Cupriavidus			Median	1.241	0.642 to 2.421	0.52	2.26	0.679 to 7.137	0.168
Helicobacter			Median	1.214	0.627 to 2.369	0.5643	1.77	0.798 to 4.075	0.1659

Cox regression for Age, Gender, localisation of tumour, UICC-classification, Laurens-classification and the abundance under or over the median for *Prevotella*, *Asinibacterium*, *Lactobacillus*, *Streptococcus*, *Fusobacterium*, Cupriavidus and *Helicobacter*.

Significant values are in bold.

**Figure 3:**
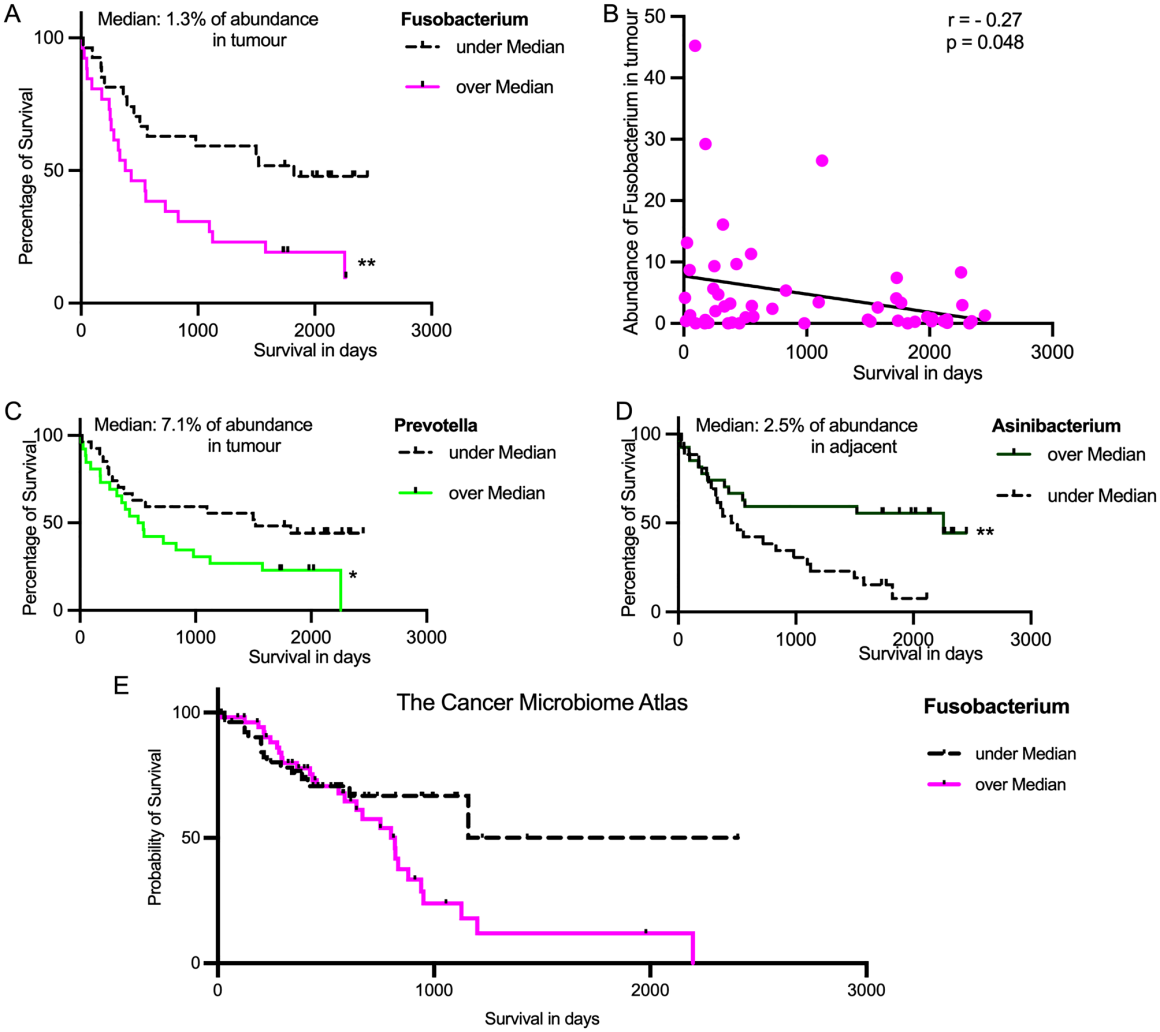
Kaplan–Meier-curve of the abundances of *Fusobacterium* in tumour tissue (**A**). Correlation of OS data (in days) with the abundance of *Fusobacterium* in tumour tissue, with linear regression and Spearman rank coefficient (**B**). Kaplan–Meier-Curves of the abundances of *Prevotella* in tumour tissue (**C**) and the abundances of *Asinibacterium* in adjacent tissue (**D**). Significant differences in OS are indicated by * if q < 0.05 and ** if q < 0.01. (**E**) Kaplan–Meier-Curve of the abundance of *Fusobacterium* based on publicly available TCMA data.

To evaluate the effect of bacterial diversity on the OS, we applied median abundance values as a cut-off for subgrouping. Neither species richness nor Shannon diversity in T-GC or NT-GC samples had any association with differences in OS in GC patients (Supplementary Fig. S3G–J). However, systematic analysis of bacterial taxa revealed that three out of eight families were associated with OS differences (Supplementary Fig. S4). Fusobacteriaceae, due to *Fusobacterium*, and Chitinophagaceae, due to *Asinibacterium*, were associated with statistically significant although discordant difference depending on the tissue type T-GC vs. NT-GC (Fig. 3). In the family Prevotellaceae, the genus *Prevotella* showed a residual association with worse OS. Higher abundance of *Fusobacterium* in T-GC was associated with a significantly worse OS (Fig. 3). *Prevotella* was associated with a trend similar to that of *Fusobacterium* but to a lesser extent (*p* = 0.045, Fig. 3C).

Neither *Fusobacterium* nor *Prevotella* were associated with differences in OS when the abundances were considered in NT-GC samples (Supplementary Fig. S5). However, patients with higher abundance of *Asinibacterium* in NT-GC had better OS (*p* = 0.0044), while no difference was found in T-GC (Fig. 3D and Supplementary Fig. S5). Since the abundance of *Fusobacterium* and *Prevotella* in adjacent and tumour tissue showed a strong positive correlation (Supplementary Fig. S5), we believe that an observed association with worse overall survival may be linked to functional role in tumorous tissues. Abundance of *H. pylori* was not associated with OS and the abundances in tumour and adjacent tissue are less correlated (Supplementary Figs. S4 and S5).

To confirm those results, we applied Cox regression analysis. In univariable Cox regression analyses, UICC III–IV (*p* = 0.04) and abundance higher than the median of *Prevotella* (*p* = 0.049) and *Fusobacterium* (*p* = 0.01) were significantly associated with the overall survival of GC patients. Multivariable analysis performed using Cox proportional hazards model revealed that diffuse type according to Lauren’s classification (*p* = 0.002) and high *Fusobacterium* abundance (*p* = 0.009) were independently predictive for the worse overall survival of patients with GC (Table 1).

To validate our findings, we accessed publicly available data from the TCMA^24^ cohort of 107 patients with a survival data up to 7 years and including 16% of diffuse type tumours (our cohort: 53 patients with 7 years survival follow-up, 50% diffuse type). The Kaplan–Meier-curve analysis based on the abundance of *Fusobacterium* revealed a trend for a worse overall survival (*p* = 0.08) partially supporting our hypothesis (Fig. 3E).

### Phylogenetic biodiversity of Fusobacterium species in the stomach

**Figure 4:**
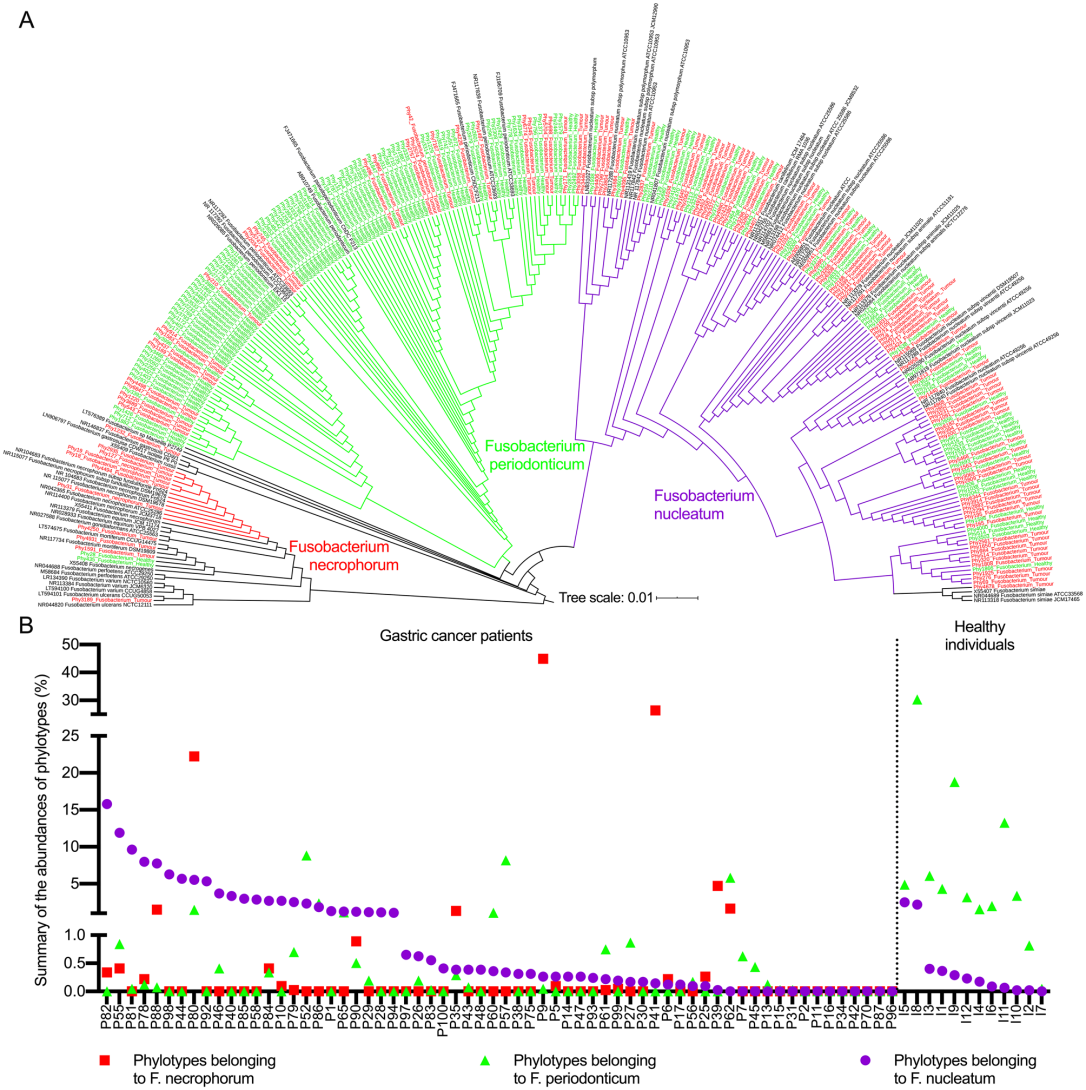
Phylogenetic tree built with the DNA sequences of the V1–V2 region of the 16S rRNA gene from 108 phylotypes annotated to *Fusobacterium* from the tumour tissue of the 64 GC patients of this study (red), 127 phylotypes annotated to *Fusobacterium* from corpus tissue of 12 healthy individuals previously published^8^ (green) and 53 type strains of *Fusobacterium* (black). Cluster in red contains sequences belonging to *F. necrophorum*, cluster in green contains sequences belonging to *F. periodonticum*, and cluster in violet contains sequences annotated as *F. nucleatum* (**A**). Percentage of abundances of *F. necrophorum*, *F. periodonticum*, and *F. nucleatum* in tumour tissues and healthy tissues (**B**).

Next, we performed a detailed characterization of *Fusobacterium* species in the stomach following a phylotype annotation. The DNA sequences of the phylotypes taxonomically annotated as *Fusobacterium* detected in the present study (n = 64, 108 sequences) were compared with publicly available data from the previous study with phylotypes annotated also as *Fusobacterium* detected in healthy individuals (n = 12, 127 sequences)^8^ and linked to 53 types strains of the genus *Fusobacterium* available in NCBI database^25^. The Supplementary Fig. S6 displays the mismatches of example 16S sequences annotated to different Fusobacterium species indicating the species resolution specially of the V1–V2 region (primers used in this work). After sequences alignment and phylogenetic tree construction, the majority of the phylotypes detected in GC patients clustered around the species *F. nucleatum* or *F. necrophorum,* whereas in healthy individuals, the vast majority of the phylotypes clustered together around the species *F. periodonticum* (Fig. 4A). Interestingly, when the abundances of the phylotypes belonging to *Fusobacterium* in both cohorts were compared, GC patients harboured higher abundances of *F. necrophorum* or *F. nucleatum* compared to healthy individuals in the stomach tissue (Fig. 4B). Furthermore, the majority of GC patients with *F. periodonticum* also had detectable *F. nucleatum* in much higher abundance. No significant difference could be found between the abundance of *F. nucleatum* in tumour and adjacent tissue of GC patients (Supplementary Fig. S7). In healthy individuals, however, *F. periodonticum* was more abundant than *F. nucleatum*. Similar analyses were performed with the phylotypes annotated as *Prevotella* and *Asinibacterium*, and no significant species differences between tumour and healthy tissues were found for those two genera (data not shown).

### Bacterial community network in GC patients

**Figure 5:**
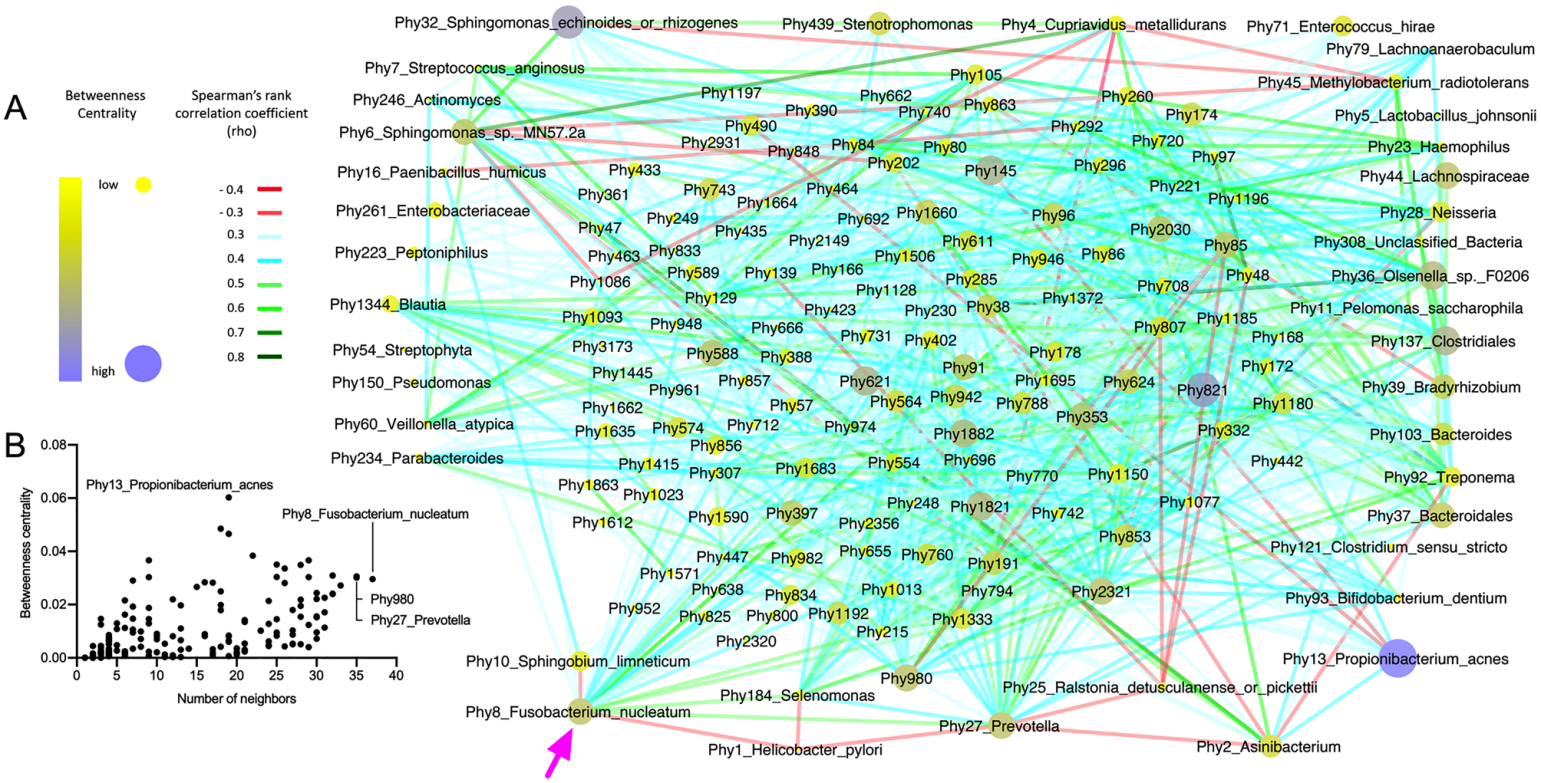
Bacterial community network with the interactions between the phylotypes in GC samples (**A**). The annotation of the phylotypes displays only if the abundance was > 10% in at least one sample. Interactions were considered statistically significant if − 0.3 < ρ > 0.3 and if q-value was < 0.05. Betweenness centrality as a function of the number of neighbours from all nodes of the bacterial community network (**B**).

To understand the bacterial interaction in GC, we performed a bacterial network analysis. The extraordinary complexity of the bacterial community in the stomach tissue became evident when the co-occurrences between phylotypes were investigated (Fig. 5 and Supplementary Table S7). A total of 1191 positive and negative co-occurrences were detected between phylotypes (Fig. 5A). *H. pylori* (Phy1) only showed three negative correlations with three phylotypes belonging to *Fusobacterium nucleatum* (Phy8), *Prevotella sp.* (Phy27) and *Selenomonas* sp*.* (Phy184). However, Phy8, Phy27, and Phy184 showed 37, 35, and 24 neighbours in the bacterial network, respectively, suggesting that *H. pylori* does not co-occur with any bacteria and even is antagonistic to *F. nucleatum* and/or *Prevotella* *sp.* In contrast to *H. pylori*, all the interactions detected for both *F. nucleatum* and *Prevotella* *sp.* were positive, with the exceptions of Phy1 and Phy10 (*Sphingobium limneticum*), which negatively co-occurred with *F. nucleatum*, as well as Phy1, Phy2 (*Asinibacterium*), and Phy25 (*Ralstonia detusculanense* or *picketii*), which negatively co-occurred with *Prevotella*. These findings suggest that *F. nucleatum* and *Prevotella* have a close interplay with each other and with stomach bacterial communities in GC patients. In fact, both Phy8 and Phy27 had the maximum number of neighbours (together with Phy980 belonging to Ruminococcaceae) in the bacterial network (Fig. 5B and Supplementary Table S7).

## Discussion

Microbial alterations have been reported for GC; however, whether the mucosal microbial community of the stomach may be linked to cancer prognosis, as shown for instance, in CRC, has not been studied yet. In this work, we systematically characterized the mucosal microbiome community in paired tumorous and adjacent tissues and evaluated potential association with prognosis in GC patients. According to our data, the abundance of *F. nucleatum* and the genus *Prevotella* in tumorous but not in adjacent tissues was associated with worse OS in GC patients, suggesting its involvement in the functional fine-tuning of molecular pathways related to tumour progression. Furthermore, *Asinibacterium* in non-tumorous adjacent tissues was associated with better OS in GC patients, suggesting that microbial environment in the stomach may also contribute to phenotype of the GC.

In comparison to various other compartments, and specifically the colon, gastric microhabitats may be quite sensitive to environmental changes including pH, drugs, diet, and others, which may become more stable along the GI tract. The gastric microbiome of the corpus and antrum has been shown to have a low level of variation^6,8,9^, while interindividual variation between subjects, as supported by our data, is quite high. The main reason for this high level of interindividual variation in overall comparison was related to *H. pylori,* as the most crucial habitat of the stomach. Nevertheless, *H. pylori* was more abundant in adjacent tissues compared to paired tumorous tissues suggesting various factors such as local pH changes, reduced mucus layer, local inflammation and likely other molecular mechanism linked to non-beneficial environment in the tumour compared to adjacent tissues. This observation was just recently confirmed using an independent set of GC tissues both at DNA and RNA level supporting our results^26^. Next to *H. pylori* also other species such as *Lactobacillus johnsonii*, *Olsenella*, *Streptococcus*, *Paenibacillus huminis*, or *Sphingomonas* were also linked to intra-individual variation. In addition, gastric tumorous tissues in comparison to non-tumorous tissues were furthermore associated with a certain shift of microbial community toward microorganisms typically found in lower GI tract such as *Bacteroides* or *Clostridium *sensu stricto^8^. Considerable changes related to GC in stomach environment may likely explain this observation which is also observed in subjects under acid-suppressive drugs. Despite this shift in microbial structure, we did not detect any clinical phenotype related to lower GI tract microbiome, but further data with larger samples size may be needed to address this question in depth.

The key aim of the work was to evaluate if bacterial composition may be linked to prognostic differences as previously reported for CRC. We systematically analyzed the impact of the entire bacterial community of the stomach on the prognosis in GC patients. While species richness and Shannon diversity showed no association with prognostic phenotype, we found that three genera were associated with OS in GC patients. High abundance of *F. nucleatum* in tumour tissue but not in adjacent mucosa was significantly associated with worse prognosis, and abundance was inversely proportional with OS time. Also, higher abundance of *Prevotella* spp*.* was associated with worse prognosis. However, in adjacent non-tumorous tissues, only *Asinibacterium* spp*.* showed an opposite effect, with high abundance associated with better OS. This indicates that bacterial community may be associated with GC prognosis and that the sampling site is crucial for appropriate assessment in GC. Importantly, *F. nucleatum*-associated OS changes were independent of advanced tumour stage similar to the previous data^23^.

To gain an additional, more precise view on phylogenetic biodiversity of *Fusobacterium* species in the stomach, we performed phylotype taxonomic annotation using the data from the present study and compared to data from a previous publicly available cohort^8^. Absolute majority of bacteria from tumour specimens clustered together around the species *F. nucleatum* or *F. necrophorum,* while in healthy individuals the vast majority of the phylotypes clustered around the species *F. periodonticum.* Although *F. periodonticum* was also detectable in GC patients, the abundance of *F. nucleatum* was much higher in GC patients, while the opposite was the case in healthy individuals. This clear association between GC and *F. nucleatum* provides not only potential diagnostic opportunities, but more specifically, the possibility of targeted antibiotic treatment or bacteriophages treatment. More than that, bacterial network analysis revealed a tight interaction between *F. nucleatum* and *Prevotella* with other stomach bacterial communities as well as a negative association with *H. pylori* infection. This may prove relevant for modification of tumour environment, for instance, the use of pro- and prebiotics.

The association of *F. nucleatum* with worse prognosis in GC is still novel, even though previously described in CRC patients^27^. The amount of *F. nucleatum* DNA in colorectal cancer tissue is associated with shorter OS of the patients and is also considered a potential prognostic biomarker^20^. Species of *Fusobacterium* were observed to have a significant positive correlation with local inflammation in CRC cases^28^. In a mouse model, *F. nucleatum* increased tumour proliferation and selectively recruited tumour infiltrating myeloid cells, which can promote tumour progression^29^. Rubinstein et. al. reported that virulence factor FadA of *F. nucleatum* promotes CRC via direct binding to E-cadherin, which inhibits tumour suppressive activity of E-cadherin^30,31^. Most intriguingly, *F. nucleatum* does not simply persist in the primary tumours but seems to be carried to metastatic niches where it can be detected and even cultivated^21^. Over the past years, multiple molecular pathways have been linked to *F. nucleatum*, and although most of the data come from the studies on CRC, these similar pathways are likely deregulated in GC as well^32^. For instance, *F. nucleatum* has been shown to enhance the adhesion of CRC cells to endothelia cells, and in this way, promotes tumour progression and metastasis through upregulation of ALPK1 and ICAM1 expression^33^. At present, the molecular pathways related to *F. nucleatum* in GC have not been explored adequately and further work is needed to unravel the molecular impact.

Considering the novelty of the data, there are also a few limitations that need to be addressed in future studies. The incidence of GC in European countries is relatively low; therefore, only a limited number of well-characterized samples were included in the study, which was further impacted during the pandemic. Furthermore, all patients were included in the study before neoadjuvant chemotherapy became standard of care in Lithuania; therefore, it was possible to obtain high-quality surgical specimens without the prior impact of chemotherapy. But at the same time, we could not obtain systematic data on the adjuvant chemotherapy, meaning that this factor could not be addressed in this work systematically. Further longitudinal studies will be required to observe the gastric bacterial community evolving during the progression and treatment of GC so that the significance of *F. nucleatum* for the entire course of the disease can be assessed. In addition, the assessment of antibiotic treatment in such cohorts will be important as it may impact not only the assessment of the bacterial community, but also potentially the prognosis of *F. nucleatum* positive tumours.

In conclusion, we demonstrate that gastric mucosal bacterial compositions are highly variable in GC patients and that *H. pylori* is the main determinant of the mucosal microbial similarity. Abundance of *F. nucleatum and Prevotella* spp*.* in tumorous tissues were associated with worse OS of GC patients, suggesting mechanistic link between tumour prognosis and bacterial composition, which was further supported by taxonomic analysis. Further studies will be necessary to explore translational relevance, including response to the classical chemotherapies and immunotherapies, and potential antibiotic therapeutic options related to *F. nucleatum* positivity.

## Methods

### Study design and sample collection

Samples were collected in the Department of Gastroenterology and Surgery at the Hospital of the Lithuanian University of Health Sciences (Kaunas, Lithuania). The study was conducted according to the principles of the Declaration of Helsinki. The Kaunas Regional Bioethics Committee (No. BE-2-10) approved the study. All patients participating in the study provided written informed consent. The collection and analysis of biological material was previously described in detail elsewhere^34,35^. Briefly, specimens from tumour (T-GC) and adjacent (NT-GC) tissue were prospectively collected during surgical interventions. The cohort consisted of samples from gastric tissue and adjacent tissue from 64 patients (128 samples). The samples were immediately snap-frozen in liquid nitrogen and placed in a -80 ºC freezer. The whole study design is visualized as a graphical abstract in Fig. 1.

### Survival analysis and tumour characterization

OS data were available from the Lithuanian Cancer Registry and the Hospital of the Lithuanian University of Health Sciences for up to 2500 days follow-up of GC patients as partly described previously^23^. The OS time was defined as the time interval between the date of GC diagnosis and the date of death. From the total cohort, survival data were available for 53 patients (83% of the cohort), including 28 men and 25 women, with a mean age of 66 ± 12 years (Table 1). The Lauren´s classification and UICC staging system were used for histological assessment of GC tumours. *H. pylori* status was analysed by *H. pylori* ELISA IgG test (Virion\Serion GmbH, Germany) for the available subset of GC patients^23^.

### DNA extraction and library preparation

DNA was extracted from frozen tissue samples, pretreated with QIAzol Lysis Reagent (Qiagen, Valencia, CA) and chloroform based on manufacturer´s recommendations^34,35^. Amplicon libraries were generated by amplifying the V1–V2 region of the 16S rRNA gene after 30 cycles PCR reaction with the 27F and 338R primers^36^ and paired-end sequencing on a MiSeq (2 × 250 bp, Illumina, Hayward, California, USA)^37^.

### Bioinformatic and statistical analyses

All fastQ files, generated after sequencing and demultiplexing, were analysed using dada2 package version 1.10.1^38^ and, as result, a unique table containing all samples with the sequence reads and abundances was generated. Samples with less than 4000 reads were discarded, and only the paired samples were left for further analysis. Thus, a total of 64 patients were considered for downstream analyses. All 128 samples were resampled to equal the smallest library size of 4153 reads using the phyloseq package^39^ provide 5450 phylotypes. Rarefaction curves as well as Shannon´s diversity and richness indices were determined using the package vegan from R^40^.

Sequence reads were assigned to a taxonomic annotation against the Ribosomal Database Project (RDP)^41^, based on the naïve Bayesian classification^42^ with a pseudo-bootstrap threshold of 80%. In addition, the first 100 more abundant phylotypes in the cohort were manually analysed against the RDP database^41^ using the Seqmatch function. Microbial communities were analysed at different phylogenetic ranks in a sequential manner, from phylum to class, order, family, genus, and phylotype. Relative abundances (expressed as percentages) were used for further analysis (Supplementary Tables S1–S6).

Univariate analyses were performed using Prism 8 (GraphPad Software), while the multivariable analyses were performed using Past3^43^. The sample-similarity matrices using Bray–Curtis algorithm were constructed with the data comprising the percentage of abundances of each of the taxonomic ranks mentioned above. Based on these resemblances, samples were hierarchically clustered (1000 bootstraps) and tested for differences between adjacent and tumour sample-groups with PERMANOVA (9999 repetitions) and ANOSIM (9999 repetitions). Each data set of interest was subjected to a normality test using the D’Agostino & Pearson omnibus. Differences in the distribution of the abundances of taxa between tumour tissue and adjacent tissue, as well as between the distinctive a priori defined groups based on the prognostic data (Table 1) were calculated by the Mann–Whitney U paired test with a 95% confidence interval by using the package ExactRankTest (version 0.8–29, 2017) from R. All the other comparisons between groups were calculated using unpaired test. All *p* values were corrected by applying the Benjamini–Hochberg false-discovery rate^44^ correction (desired FDR = 5%), and the abundance of taxa between the groups was considered significantly different if the corrected *p* value (*q* value) was below 0.05.

Kaplan–Meier curves were generated in Prism8 (GraphPad software) for the age, gender, tumour location, tumour classification (UICC and Lauren´s), and for correlating the survival data of the patients with their bacterial composition either in tumour or adjacent tissues. Kaplan–Meier curves were generated with the most abundant taxa at taxonomic rank of family, firstly considering only the families more abundant than 10% in at least 5 samples and secondly, considering all the genera belonging to the families in which an increase of mortality was observed. Kaplan–Meier curves compared the survival between patients with lower abundance (in %) than the median of the correspondent taxa and those patients with higher abundance (in %) than the median. Log-rank of Mantel-Cox with a 95% confidence interval was used to determine statistical differences between the Kaplan–Meier curves.

Heatmaps were generated with the packages gplots (version 3.0.3) and RColorBrewer (version 1.1–2) from R. Phylogenetic trees were built from the similarity matrix of the sequences of DNA from the region V1–V2 in the 16S rRNA gene. The multiple sequence alignments were carried out in SeaView^45^ using the Clustal distance methods option with 1000 bootstraps. Trees were visualised with Interactive Tree of Life, iTol^46^. The bacteria community network was generated based on Spearman’s rank correlation coefficient (rho) between those phylotypes detected in at least one sample with > 1% of abundance. The Spearman correlation coefficient was calculated using the packages psych (version 1.9.12.31) and reshape2 (version 1.4.4) from R and were corrected by applying Benjamini–Hochberg false-discovery rate correction. Correlations were considered significant if −0.3 ≥ Rho ≥ 0.3 and *q* value < 0.05. Network was visualised in Cytoscape (version 3.8.0)^47^ displaying the betweenness centrality of each node.

### Data from the cancer microbiome atlas (TCMA)

To validate our results, we accessed data publicly available from the TCMA^24^ and selected sample from primary gastric cancer tissue with a survival follow up to 7 years (like in our cohort), resulting in a cohort of 107 patients (https://tcma.pratt.duke.edu/, abundance and metadata uploaded to the atlas at the 29.09.2020, accessed at the 27.09.2022). Kaplan–Meier-Curves were generated based on the abundance of *Fusobacterium*.

## Supplementary Information

Supplementary Information 1.

Supplementary Information 2.

## Data Availability

All data are included in the supplementary material.
