# Supplementary material for: Microbial composition of tumorous and adjacent gastric tissue is associated with prognosis of gastric cancer
Source: Sci Rep. 2023 Mar 21;13:4640. doi: 10.1038/s41598-023-31740-3 (PMC10030820; doi:10.1038/s41598-023-31740-3)
Supplement: Supplementary file 1 — Supplementary Information 1. [file 41598_2023_31740_MOESM1_ESM.pdf]

Microbial composition of tumorous and adjacent gastric tissue is associated with prognosis of gastric cancer

Konrad Lehr[1], Darja Nikitina[2], Ramiro Vilchez-Vargas[1], Ruta Steponaitiene [2], Cosima Thon[1], Jurgita Skieceviciene[2], Denny Schanze[3], Martin Zenker[3], Peter Malfertheiner[1,4], Juozas Kupcinskas[2,5], Alexander Link[1]

1 Department of Gastroenterology, Hepatology and Infectious Diseases, Otto-von-Guericke University Magdeburg, Germany

2 Institute for Digestive Research, Lithuanian University of Health Sciences Kaunas, Lithuania

3 Institute of Human Genetics, Otto-von-Guericke University Magdeburg, Germany

4 Department of Internal Medicine 2, University Hospital, LMU Munich, Munich, Germany

5 Department of Gastroenterology, Lithuanian University of Health Sciences Kaunas, Lithuania

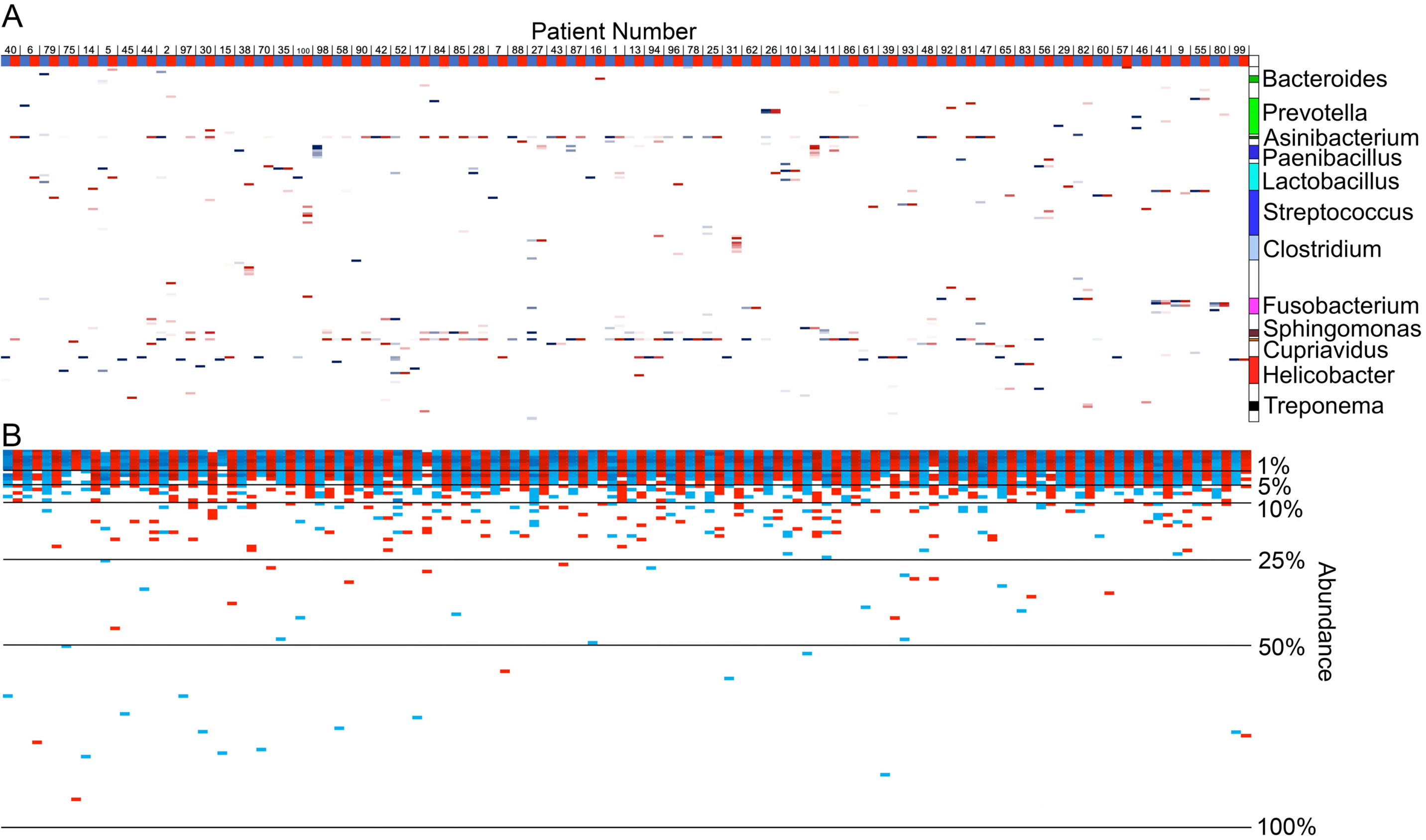

Supplementary Figure S1: Heatmap containing the phylotypes detected with >5% of abundance in tumour tissue (red) and adjacent tissue (blue). Samples are ordered in increasing % of Bray-Curtis similarity between paired samples of the same patient, and only the genera of the phylotypes found in more than 10 samples are displayed. Light red and light blue denote low abundance, while dark red and dark blue denote high abundances (A). Heatmap with the phylotype frequency per sample at a certain % of abundance (B). Samples are displayed in the same order as in A.

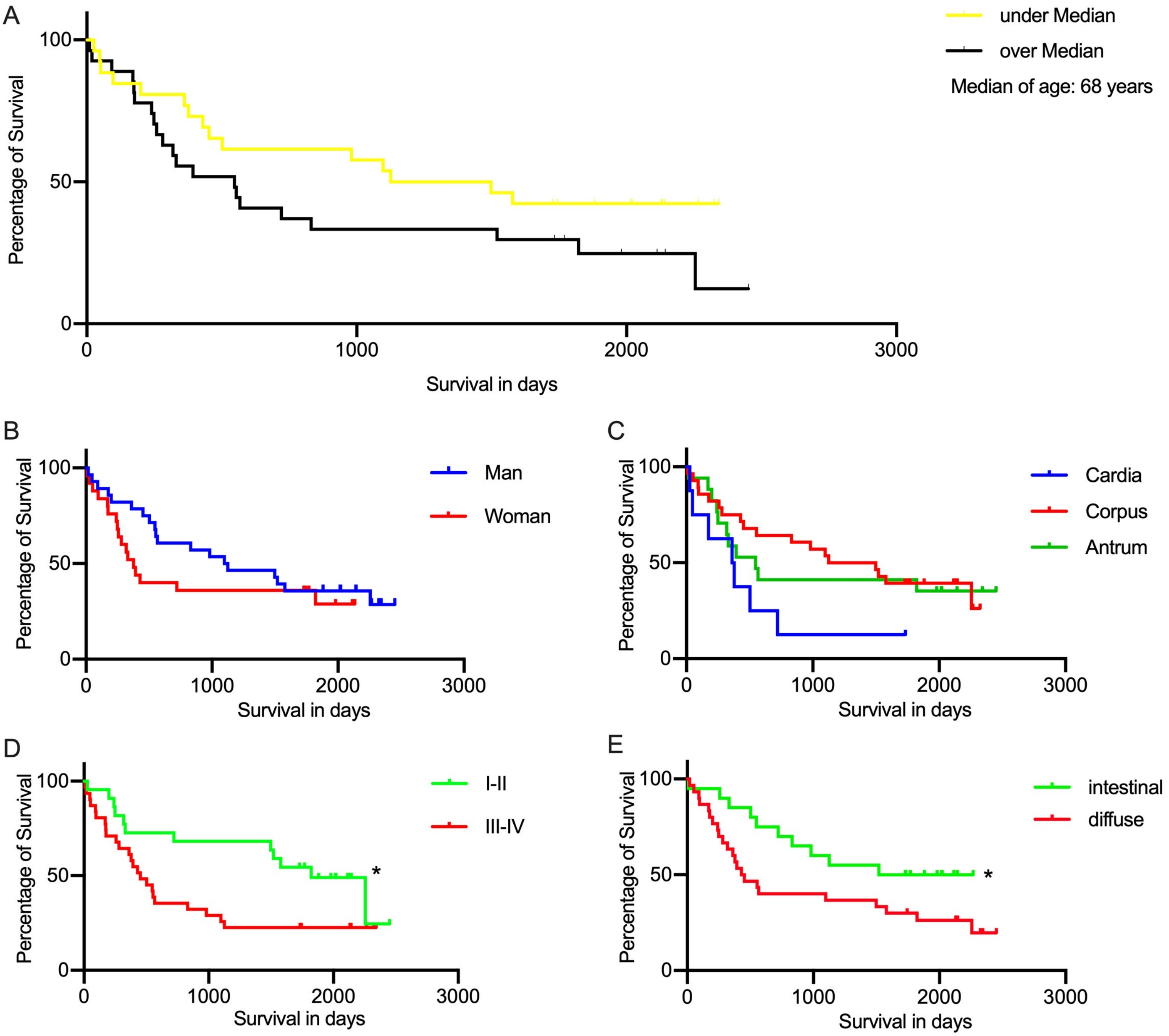

Supplementary Figure S2: Kaplan-Meier-Curves for the age (A), the gender (B), the location of tumour (C), the UICC-classification (D), and the Lauren classification (E). Significant differences in OS are indicated by * if $q < 0.05$.

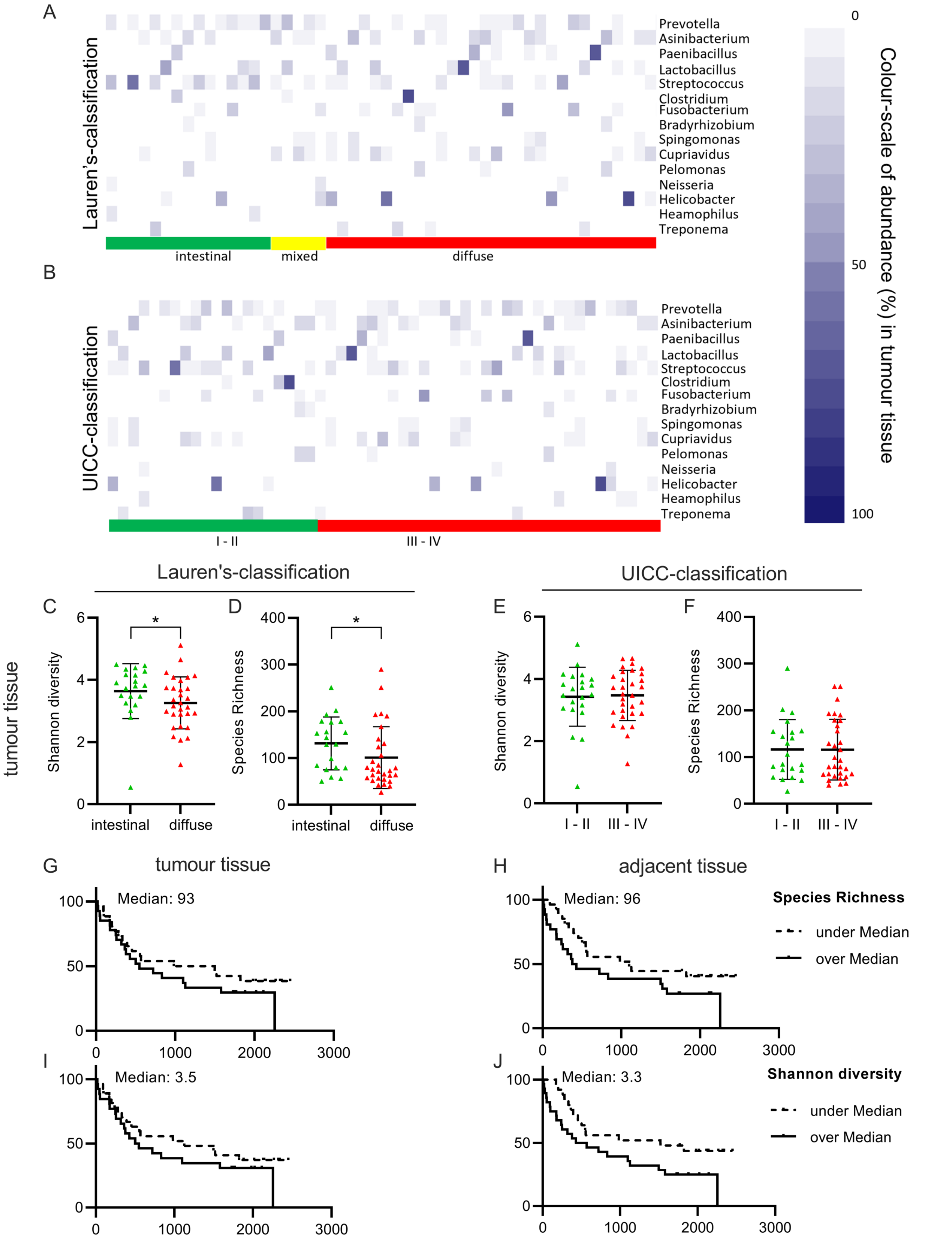

Supplementary Figure S3: Abundances of the genera grouped according to Lauren's classification (A) and UICC classification (B) of 53 GC patients. Only the genera with more abundance than 10% were considered. Shannon diversity index (C, E) and species richness (D, F) in samples with different tumour stages, according to Lauren´s classification and UICC classification. Significant differences between groups are indicated by * if $q < 0.05$. Kaplan-Meier-Curves for Shannon Index and species richness in tumour and adjacent tissues samples (G-J).

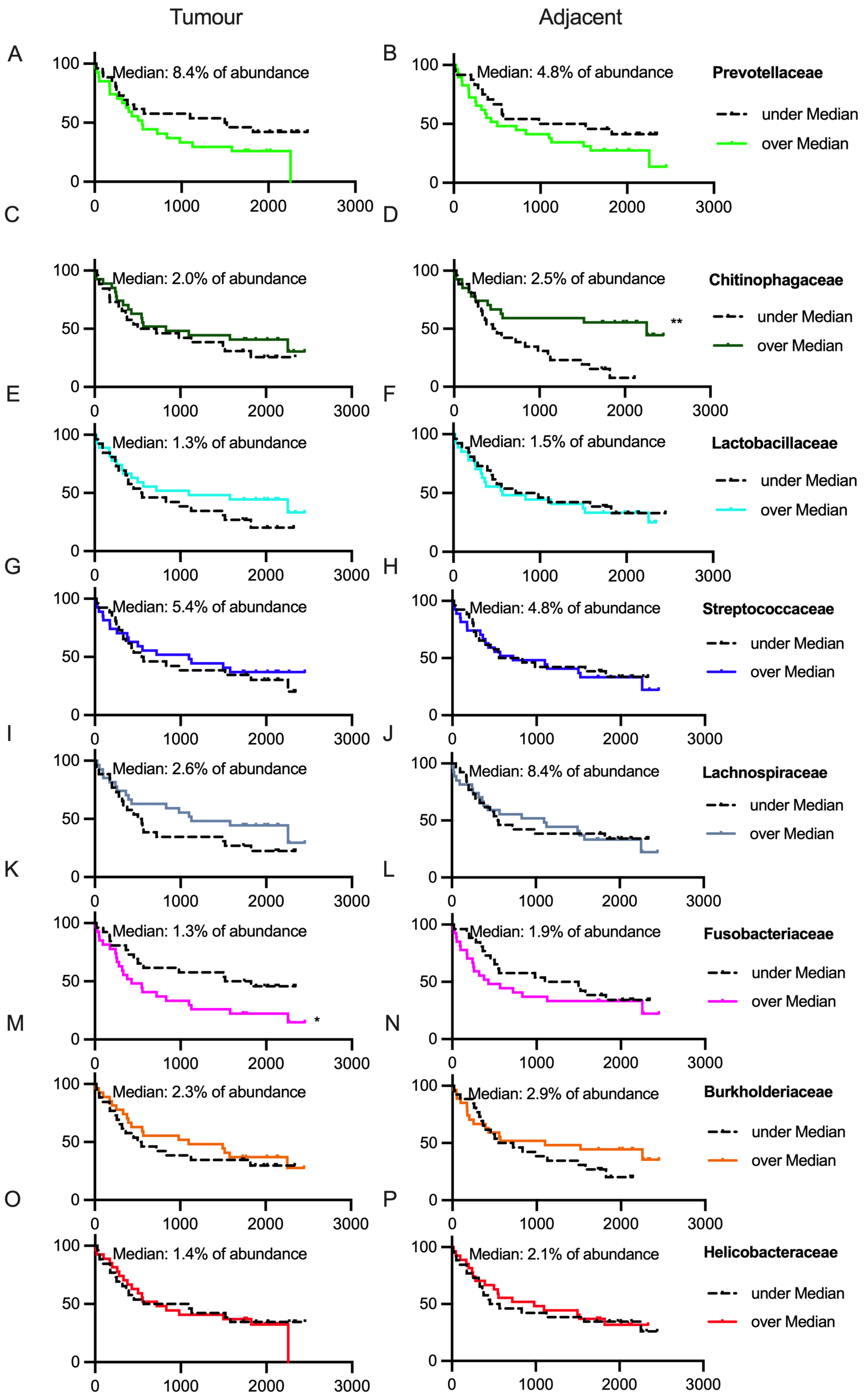

Supplementary Figure S4: Kaplan-Meier-Curves for Prevotellaceae (A-B), Chitonophagaceae (C-D), Lactobacillaceae (E-F), Streptococcaceae (G-H), Lachnospiraceae (I-J), Fusobacteriaceae (K-L), Burkholderiaceae (M-N), and Helicobacteraceae (O-P) in tumour or adjacent tissues. X-axes shows the OS in days and y-axes the percentage of OS.

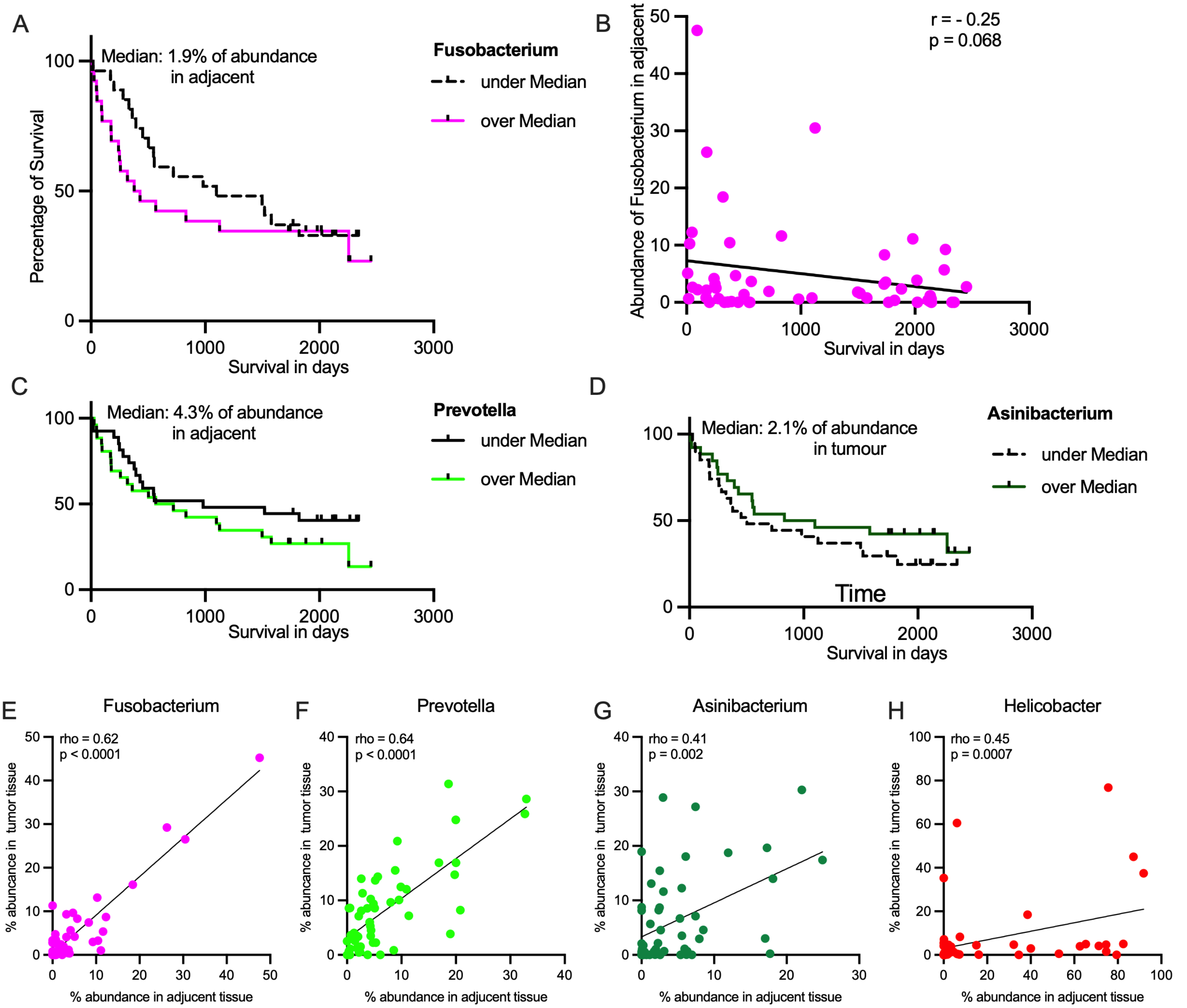

Supplementary Figure S5: Kaplan-Meier-Curves of the abundances of *Fusobacterium* in adjacent tissue (A). Correlation of OS data (in days) with the abundance of *Fusobacterium* in adjacent tissue, with linear regression and Spearman rank coefficient (B). Kaplan-Meier-Curves of the abundances of *Prevotella* in adjacent tissue (C) and the abundances of *Asinibacterium* in tumour tissue (D). X-axes show survival data in days. Significant differences in survival are indicated by * if q < 0.05 and ** if q < 0.01. Correlation of the abundance of *Fusobacterium* (E), *Prevotella* (F), *Asinibacterium* (G), *Helicobacter* (H) in tumour and adjacent tissue.

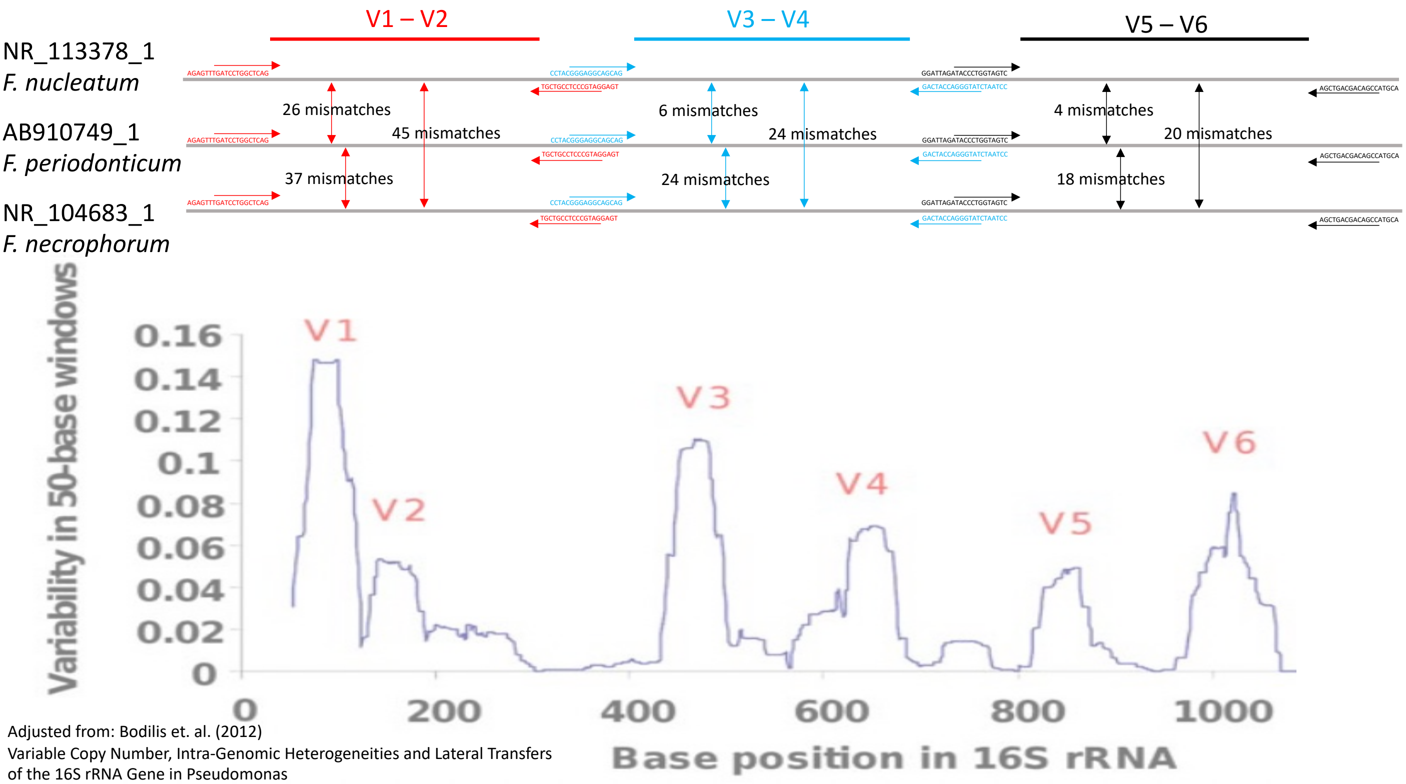

Adjusted from: Bodilis et. al. (2012)
Variable Copy Number, Intra-Genomic Heterogeneities and Lateral Transfers of the 16S rRNA Gene in Pseudomonas

Supplementary Figure S6 continues next page

```
                                            1
V1_V2F                                      AGAGTTTGAT CCTGGCTCAG ---------- ----------
V1_V2R                                      ---------- ---------- ---------- ----------
V3_V4F                                      ---------- ---------- ---------- ----------
V3_V4R                                      ---------- ---------- ---------- ----------
V5_V6F                                      ---------- ---------- ---------- ----------
V5_V6R                                      ---------- ---------- ---------- ----------
NR_113378_1_Fusobacterium_nucleatum         AGAGTTTGAT CCTGGCTCAG GATGAACGCT GACAGAATGC
AB910749_1_Fusobacterium_periodonticum      AGAGTTTGAT CCTGGCTCAG GATGAACGCT GACAGAATGC
NR_104683_1_Fusobacterium_necrophorum       AGAGTTTGAT CCTGGCTCAG GATGAACGCT GACAGAATGC

                                           41
V1_V2F                                      ---------- ---------- ---------- ----------
V1_V2R                                      ---------- ---------- ---------- ----------
V3_V4F                                      ---------- ---------- ---------- ----------
V3_V4R                                      ---------- ---------- ---------- ----------
V5_V6F                                      ---------- ---------- ---------- ----------
V5_V6R                                      ---------- ---------- ---------- ----------
NR_113378_1_Fusobacterium_nucleatum         TTAACACATG CAAGTCAACT TGAATTTGGG TTTTTAACTT
AB910749_1_Fusobacterium_periodonticum      TTAACACATG CAAGTCTACT TGAACT---- ---------T
NR_104683_1_Fusobacterium_necrophorum       TTAACACATG CAAGTCGACT CGAGTCT--- --------TC

                                           81
V1_V2F                                      ---------- ---------- ---------- ----------
V1_V2R                                      ---------- ---------- ---------- ----------
V3_V4F                                      ---------- ---------- ---------- ----------
V3_V4R                                      ---------- ---------- ---------- ----------
V5_V6F                                      ---------- ---------- ---------- ----------
V5_V6R                                      ---------- ---------- ---------- ----------
NR_113378_1_Fusobacterium_nucleatum         AGATTTGGGT GGCGGACGGG TGAGTAACGC GTAAAGAACT
AB910749_1_Fusobacterium_periodonticum      CGGTTTGGGT GGCGGACGGG TGAGTAACGC GTAAAGAACT
NR_104683_1_Fusobacterium_necrophorum       GGACTTGGGT GGCGCACGGG TGAGTAACGC GTAAAGAACT

                                          121
V1_V2F                                      ---------- ---------- ---------- ----------
V1_V2R                                      ---------- ---------- ---------- ----------
V3_V4F                                      ---------- ---------- ---------- ----------
V3_V4R                                      ---------- ---------- ---------- ----------
V5_V6F                                      ---------- ---------- ---------- ----------
V5_V6R                                      ---------- ---------- ---------- ----------
NR_113378_1_Fusobacterium_nucleatum         TGCCTCACAG TTAGGGACAA CATTTAGAAA TGAATGCTAA
AB910749_1_Fusobacterium_periodonticum      TGCCTCACAG TTAGGGACAA CATTTGGAAA CGAATGCTAA
NR_104683_1_Fusobacterium_necrophorum       TGCCTCTTAG ACCGGGACAA CATCTGGAAA CGGATGCTAA

                                          161
V1_V2F                                      ---------- ---------- ---------- ----------
V1_V2R                                      ---------- ---------- ---------- ----------
V3_V4F                                      ---------- ---------- ---------- ----------
V3_V4R                                      ---------- ---------- ---------- ----------
V5_V6F                                      ---------- ---------- ---------- ----------
V5_V6R                                      ---------- ---------- ---------- ----------
NR_113378_1_Fusobacterium_nucleatum         TACCTGATAT TATGATTTTA AGGCATCTTA GAATTATGAA
AB910749_1_Fusobacterium_periodonticum      TACCTGATAT TATGATTTTA GGGCATCCTA AGATTATGAA
NR_104683_1_Fusobacterium_necrophorum       TACCGGATAT TATGGTTTTT TCGCATGGAG GAATCATGAA

                                          201
V1_V2F                                      ---------- ---------- ---------- ----------
V1_V2R                                      ---------- ---------- ---------- ----------
V3_V4F                                      ---------- ---------- ---------- ----------
V3_V4R                                      ---------- ---------- ---------- ----------
V5_V6F                                      ---------- ---------- ---------- ----------
V5_V6R                                      ---------- ---------- ---------- ----------
NR_113378_1_Fusobacterium_nucleatum         AGCTATAAGC ACTGTGAGAG AGCTTTGCGT CCCATTAGCT
AB910749_1_Fusobacterium_periodonticum      AGCTATATGC GCTGTGAGAG AGCTTTGCGT CCCATTAGCT
NR_104683_1_Fusobacterium_necrophorum       AGCTAGATGC GCTAAGAGAG AGCTTTGCGT CCCATTAGCT

                                          241
V1_V2F                                      ---------- ---------- ---------- ----------
V1_V2R                                      ---------- ---------- ---------- ----------
V3_V4F                                      ---------- ---------- ---------- ----------
V3_V4R                                      ---------- ---------- ---------- ----------
V5_V6F                                      ---------- ---------- ---------- ----------
V5_V6R                                      ---------- ---------- ---------- ----------
NR_113378_1_Fusobacterium_nucleatum         AGTTGGAGAG GTAACAGCTC ACCAAGGCGA TGATGGGTAG
AB910749_1_Fusobacterium_periodonticum      AGTTGGAGAG GTAACGGCTC ACCAAGGCGA TGATGGGTAG
NR_104683_1_Fusobacterium_necrophorum       AGTTGGTGAG GTAACGGCCC ACCAAGGCAA TGATGGGTAG
```

Supplementary Figure S6 continues next page

```
                                          281
V1_V2F                                      ---------- ---------- ---------- ----------
V1_V2R                                      ---------- ---------- ---------- ----------
V3_V4F                                      ---------- ---------- ---------- ----------
V3_V4R                                      ---------- ---------- ---------- ----------
V5_V6F                                      ---------- ---------- ---------- ----------
V5_V6R                                      ---------- ---------- ---------- ----------
NR_113378_1_Fusobacterium_nucleatum         CCGGCCTGAG AGGGTGAACG GCCACAAGGG GACTGAGACA
AB910749_1_Fusobacterium_periodonticum      CCGGCCTGAG AGGGTGAACG GCCACAAGGG GACTGAGACA
NR_104683_1_Fusobacterium_necrophorum       CCGGCCTGAG AGGGTGAACG GCCACAAGGG GACTGAGACA

                                          321
V1_V2F                                      ---------- ---------- ---------- ----------
V1_V2R                                      --------AC TCCTACGGGA GGCAGCA--- ----------
V3_V4F                                      ---------- -CCTACGGGA GGCAGCAG-- ----------
V3_V4R                                      ---------- ---------- ---------- ----------
V5_V6F                                      ---------- ---------- ---------- ----------
V5_V6R                                      ---------- ---------- ---------- ----------
NR_113378_1_Fusobacterium_nucleatum         CGGCCCTTAC TCCTACGGGA GGCAGCAGTG GGGAATATTG
AB910749_1_Fusobacterium_periodonticum      CGGCCCTTAC TCCTACGGGA GGCAGCAGTG GGGAATATTG
NR_104683_1_Fusobacterium_necrophorum       CGGCCCTTAC TCCTACGGGA GGCAGCAGTG GGGAATATTG

                                          361
V1_V2F                                      ---------- ---------- ---------- ----------
V1_V2R                                      ---------- ---------- ---------- ----------
V3_V4F                                      ---------- ---------- ---------- ----------
V3_V4R                                      ---------- ---------- ---------- ----------
V5_V6F                                      ---------- ---------- ---------- ----------
V5_V6R                                      ---------- ---------- ---------- ----------
NR_113378_1_Fusobacterium_nucleatum         GACAATGGAC CGAGAGTCTG ATCCAGCAAT TCTGTGTGCA
AB910749_1_Fusobacterium_periodonticum      GACAATGGAC CAAGAGTCTG ATCCAGCAAT TCTGTGTGCA
NR_104683_1_Fusobacterium_necrophorum       GACAATGGAC CACAAGTCTG ATCCAGCAAT TCTGTGTGCA

                                          401
V1_V2F                                      ---------- ---------- ---------- ----------
V1_V2R                                      ---------- ---------- ---------- ----------
V3_V4F                                      ---------- ---------- ---------- ----------
V3_V4R                                      ---------- ---------- ---------- ----------
V5_V6F                                      ---------- ---------- ---------- ----------
V5_V6R                                      ---------- ---------- ---------- ----------
NR_113378_1_Fusobacterium_nucleatum         CGATGAAGTT TTTCGGAATG TAAAGTGCTT TCAGTTGGGA
AB910749_1_Fusobacterium_periodonticum      CGATGACGTT TTTCGGAATG TAAAGTGCTT TCAGTTGGGA
NR_104683_1_Fusobacterium_necrophorum       CGATGACGTT TTTCGGAATG TAAAGTGCTT TCAGTCGGGA

                                          441
V1_V2F                                      ---------- ---------- ---------- ----------
V1_V2R                                      ---------- ---------- ---------- ----------
V3_V4F                                      ---------- ---------- ---------- ----------
V3_V4R                                      ---------- ---------- ---------- ----------
V5_V6F                                      ---------- ---------- ---------- ----------
V5_V6R                                      ---------- ---------- ---------- ----------
NR_113378_1_Fusobacterium_nucleatum         AGAAAGAAAT GACGGTACCA ACAGAAGAAG TGACGGCTAA
AB910749_1_Fusobacterium_periodonticum      AGAAAAAAAT GACGGTACCA ACAGAAGAAG TGACGGCTAA
NR_104683_1_Fusobacterium_necrophorum       AGAAGTCAGT GACGGTACCG ACAGAAGAAG CGACGGCTAA

                                          481
V1_V2F                                      ---------- ---------- ---------- ----------
V1_V2R                                      ---------- ---------- ---------- ----------
V3_V4F                                      ---------- ---------- ---------- ----------
V3_V4R                                      ---------- ---------- ---------- ----------
V5_V6F                                      ---------- ---------- ---------- ----------
V5_V6R                                      ---------- ---------- ---------- ----------
NR_113378_1_Fusobacterium_nucleatum         ATACGTGCCA GCAGCCGCGG TAATACGTAT GTCACGAGCG
AB910749_1_Fusobacterium_periodonticum      ATACGTGCCA GCAGCCGCGG TAATACGTAT GTCACAAGCG
NR_104683_1_Fusobacterium_necrophorum       ATACGTGCCA GCAGCCGCGG TAATACGTAT GTCGCAAGCG

                                          521
V1_V2F                                      ---------- ---------- ---------- ----------
V1_V2R                                      ---------- ---------- ---------- ----------
V3_V4F                                      ---------- ---------- ---------- ----------
V3_V4R                                      ---------- ---------- ---------- ----------
V5_V6F                                      ---------- ---------- ---------- ----------
V5_V6R                                      ---------- ---------- ---------- ----------
NR_113378_1_Fusobacterium_nucleatum         TTATCCGGAT TTATTGGGCG TAAAGCGCGT CTAGGTGGTT
AB910749_1_Fusobacterium_periodonticum      TTATCCGGAT TTATTGGGCG TAAAGCGCGT CTAGGTGGTT
NR_104683_1_Fusobacterium_necrophorum       TTATCCGGAT TTATTGGGCG TAAAGCGCGT CTAGGCGGCA
```

Supplementary Figure S6 continues next page

```
                                          561
V1_V2F                                      ---------- ---------- ---------- ----------
V1_V2R                                      ---------- ---------- ---------- ----------
V3_V4F                                      ---------- ---------- ---------- ----------
V3_V4R                                      ---------- ---------- ---------- ----------
V5_V6F                                      ---------- ---------- ---------- ----------
V5_V6R                                      ---------- ---------- ---------- ----------
NR_113378_1_Fusobacterium_nucleatum         ATGTAAGTCT GATGTGAAAA TGCAGGGCTC AACTCTGTAT
AB910749_1_Fusobacterium_periodonticum      ATGTAAGTCT GATGTGAAAA TGCAGGGCTC AACTCTGTAT
NR_104683_1_Fusobacterium_necrophorum       AGGAAAGTCT GATGTGAAAA TGCGGAGCTC AACTCCGTAT

                                          601
V1_V2F                                      ---------- ---------- ---------- ----------
V1_V2R                                      ---------- ---------- ---------- ----------
V3_V4F                                      ---------- ---------- ---------- ----------
V3_V4R                                      ---------- ---------- ---------- ----------
V5_V6F                                      ---------- ---------- ---------- ----------
V5_V6R                                      ---------- ---------- ---------- ----------
NR_113378_1_Fusobacterium_nucleatum         TGCGTTGGAA ACTGTATAAC TAGAGTACTG GAGAGGTAAG
AB910749_1_Fusobacterium_periodonticum      TGCGTTGGAA ACTGCATGAC TAGAGTACTG GAGAGGTAAG
NR_104683_1_Fusobacterium_necrophorum       GGCGTTGGAA ACTGCCTTAC TAGAGTACTG GAGAGGTAGG

                                          641
V1_V2F                                      ---------- ---------- ---------- ----------
V1_V2R                                      ---------- ---------- ---------- ----------
V3_V4F                                      ---------- ---------- ---------- ----------
V3_V4R                                      ---------- ---------- ---------- ----------
V5_V6F                                      ---------- ---------- ---------- ----------
V5_V6R                                      ---------- ---------- ---------- ----------
NR_113378_1_Fusobacterium_nucleatum         CGGAACTACA AGTGTAGAGG TGAAATTCGT AGATATTTGT
AB910749_1_Fusobacterium_periodonticum      CGGAACTACA AGTGTAGAGG TGAAATTCGT AGATATTTGT
NR_104683_1_Fusobacterium_necrophorum       CGGAACTACA AGTGTAGAGG TGAAATTCGT AGATATTTGT

                                          681
V1_V2F                                      ---------- ---------- ---------- ----------
V1_V2R                                      ---------- ---------- ---------- ----------
V3_V4F                                      ---------- ---------- ---------- ----------
V3_V4R                                      ---------- ---------- ---------- ----------
V5_V6F                                      ---------- ---------- ---------- ----------
V5_V6R                                      ---------- ---------- ---------- ----------
NR_113378_1_Fusobacterium_nucleatum         AGGAATGCCG ATGGGGAAGC CAGCTTACTG GACAGATACT
AB910749_1_Fusobacterium_periodonticum      AGGAATGCCG ATGGGGAAGC CAGCTTACTG GACAGATACT
NR_104683_1_Fusobacterium_necrophorum       AGGAATGCCG ATGGGGAAGC CAGCCTACTG GACAGATACT

                                          721
V1_V2F                                      ---------- ---------- ---------- ----------
V1_V2R                                      ---------- ---------- ---------- ----------
V3_V4F                                      ---------- ---------- ---------- ----------
V3_V4R                                      ---------- ---------- ---------- ---GGATTAG
V5_V6F                                      ---------- ---------- ---------- ---GGATTAG
V5_V6R                                      ---------- ---------- ---------- ----------
NR_113378_1_Fusobacterium_nucleatum         GACGCTAAAG CGCGAAAGCG TGGGTAGCAA ACAGGATTAG
AB910749_1_Fusobacterium_periodonticum      GACGCTAAAG CGCGAAAGCG TGGGTAGCAA ACAGGATTAG
NR_104683_1_Fusobacterium_necrophorum       GACGCTAAAG CGCGAAAGCG TGGGTAGCAA ACAGGATTAG

                                          761
V1_V2F                                      ---------- ---------- ---------- ----------
V1_V2R                                      ---------- ---------- ---------- ----------
V3_V4F                                      ---------- ---------- ---------- ----------
V3_V4R                                      ATACCCTGGT AGTC------ ---------- ----------
V5_V6F                                      ATACCCTGGT AGTC------ ---------- ----------
V5_V6R                                      ---------- ---------- ---------- ----------
NR_113378_1_Fusobacterium_nucleatum         ATACCCTGGT AGTCCACGCT GTAAACGATG ATTACTAGGT
AB910749_1_Fusobacterium_periodonticum      ATACCCTGGT AGTCCACGCC GTAAACGATG ATTACTAGGT
NR_104683_1_Fusobacterium_necrophorum       ATACCCTGGT AGTCCACGCT GTAAACGATG ATTACTAGGT

                                          801
V1_V2F                                      ---------- ---------- ---------- ----------
V1_V2R                                      ---------- ---------- ---------- ----------
V3_V4F                                      ---------- ---------- ---------- ----------
V3_V4R                                      ---------- ---------- ---------- ----------
V5_V6F                                      ---------- ---------- ---------- ----------
V5_V6R                                      ---------- ---------- ---------- ----------
NR_113378_1_Fusobacterium_nucleatum         GTTGGGGGTC GAACCTCAGC GCCCAAGCAA ACGCGATAAG
AB910749_1_Fusobacterium_periodonticum      GTTGGGGGTC GAACCTCAGC GCCCAAGCTA ACGCGATAAG
NR_104683_1_Fusobacterium_necrophorum       GTTGGGGGTC AAACCTCAGC GCCCAAGCTA ACGCGATAAG
```

Supplementary Figure S6 continues next page

```
                                            841
V1_V2F                                        ---------- ---------- ---------- ----------
V1_V2R                                        ---------- ---------- ---------- ----------
V3_V4F                                        ---------- ---------- ---------- ----------
V3_V4R                                        ---------- ---------- ---------- ----------
V5_V6F                                        ---------- ---------- ---------- ----------
V5_V6R                                        ---------- ---------- ---------- ----------
NR_113378_1_Fusobacterium_nucleatum           TAATCCGCCT GGGGAGTACG TACGCAAGTA TGAAACTCAA
AB910749_1_Fusobacterium_periodonticum        TAATCCGCCT GGGGAGTACG TACGCAAGTA TGAAACTCAA
NR_104683_1_Fusobacterium_necrophorum         TAATCCGCCT GGGGAGTACG TACGCAAGTA TGAAACTCAA

                                            881
V1_V2F                                        ---------- ---------- ---------- ----------
V1_V2R                                        ---------- ---------- ---------- ----------
V3_V4F                                        ---------- ---------- ---------- ----------
V3_V4R                                        ---------- ---------- ---------- ----------
V5_V6F                                        ---------- ---------- ---------- ----------
V5_V6R                                        ---------- ---------- ---------- ----------
NR_113378_1_Fusobacterium_nucleatum           AGGAATTGAC GGGGACCCGC ACAAGCGGTG GAGCATGTGG
AB910749_1_Fusobacterium_periodonticum        AGGAATTGAC GGGGACCCGC ACAAGCGGTG GAGCATGTGG
NR_104683_1_Fusobacterium_necrophorum         AGGAATTGAC GGGGACCCGC ACAAGCGGTG GAGCATGTGG

                                            921
V1_V2F                                        ---------- ---------- ---------- ----------
V1_V2R                                        ---------- ---------- ---------- ----------
V3_V4F                                        ---------- ---------- ---------- ----------
V3_V4R                                        ---------- ---------- ---------- ----------
V5_V6F                                        ---------- ---------- ---------- ----------
V5_V6R                                        ---------- ---------- ---------- ----------
NR_113378_1_Fusobacterium_nucleatum           TTTAATTCGA CGCAACGCGA GGAACCTTAC CAGCGTTTGA
AB910749_1_Fusobacterium_periodonticum        TTTAATTCGA CGCAACGCGA GGAACCTTAC CAGCGTTTGA
NR_104683_1_Fusobacterium_necrophorum         TTTAATTCGA CGCAACGCGA GGAACCTTAC CAGCGTTTGA

                                            961
V1_V2F                                        ---------- ---------- ---------- ----------
V1_V2R                                        ---------- ---------- ---------- ----------
V3_V4F                                        ---------- ---------- ---------- ----------
V3_V4R                                        ---------- ---------- ---------- ----------
V5_V6F                                        ---------- ---------- ---------- ----------
V5_V6R                                        ---------- ---------- ---------- ----------
NR_113378_1_Fusobacterium_nucleatum           CATCTTAGGA ATGAGATAGA GATATTTCAG TGTCCCTTCG
AB910749_1_Fusobacterium_periodonticum        CATCTTAGGA ATGAGACAGA GATGTTTCAG TGTCCCTTCG
NR_104683_1_Fusobacterium_necrophorum         CATCCTACGA ACGGAGCAGA GATGCGCCGG TGCCCTTTCG

                                           1001
V1_V2F                                        ---------- ---------- ---------- ----------
V1_V2R                                        ---------- ---------- ---------- ----------
V3_V4F                                        ---------- ---------- ---------- ----------
V3_V4R                                        ---------- ---------- ---------- ----------
V5_V6F                                        ---------- ---------- ---------- ----------
V5_V6R                                        ---------- ---------- -TGCATGGCT GTCGTCAGCT
NR_113378_1_Fusobacterium_nucleatum           GGGAAACCTA AAGACAGGTG GTGCATGGCT GTCGTCAGCT
AB910749_1_Fusobacterium_periodonticum        GGGAAACCTA AAGACAGGTG GTGCATGGCT GTCGTCAGCT
NR_104683_1_Fusobacterium_necrophorum         GGGGAACGTA GTGACAGGTG GTGCATGGCT GTCGTCAGCT
```

Supplementary Figure S6: Analysis of the species resolution in *Fusobacterium* using different regions of the 16S rRNA gene.

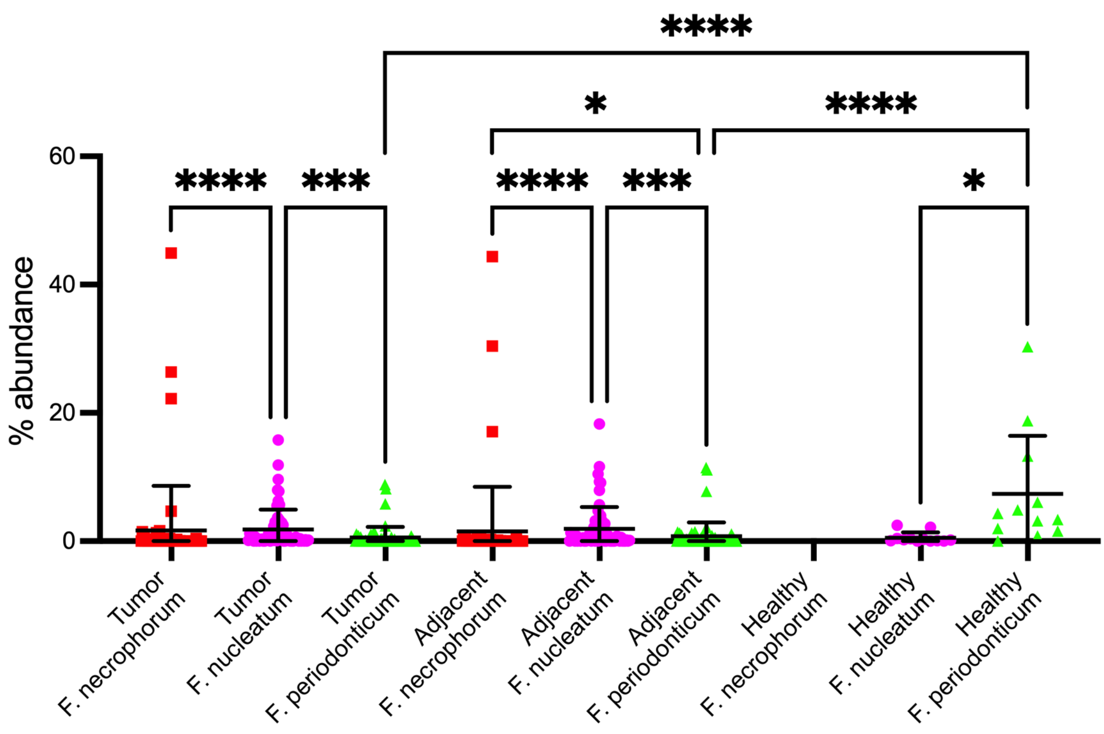

Supplementary Figure S7: Abundance of *Fusobacterium* species in tumour, adjacent and healthy tissue.
